# Changes in perception of treatment efficacy are associated to the magnitude of the nocebo effect and to personality traits

**DOI:** 10.1038/srep30671

**Published:** 2016-07-29

**Authors:** Nicole Corsi, Mehran Emadi Andani, Michele Tinazzi, Mirta Fiorio

**Affiliations:** 1 Department of Neurosciences, Biomedicine and Movement Sciences, University of Verona, Verona, 37131, Italy; 2 Department of Pain and Translational Symptom Science, University of Maryland Baltimore, Baltimore, 21201, USA; 3 Department of Biomedical Engineering, University of Isfahan, Isfahan, 81746, Iran

## Abstract

The nocebo effect in motor performance consists in a reduction of force and increase of fatigue following the application of an inert treatment that the recipient believes to be effective. This effect is variable across individuals and it is usually stronger if conditioning –exposure to the active effect of the treatment– precedes a test session, in which the treatment is inert. In the current explorative study we used a conditioning procedure to investigate whether subjective perception of treatment effectiveness changes between the conditioning and the test session and whether this change is related to dispositional traits and to the nocebo-induced reduction of force. Results showed that 56.1% of participants perceived the treatment as more effective in the test than in the conditioning session, had a more pronounced reduction of force, felt more effort and sense of weakness and were characterized by lower levels of optimism and higher anxiety traits compared to the other 43.9% of participants, who conversely perceived the treatment as less effective in the test session than in the conditioning. These findings highlight for the first time a link between changes in perception of treatment effectiveness, personality traits and the magnitude of the nocebo response in motor performance.

The nocebo effect can be defined as a negative outcome following the application of an inert treatment that the recipient believes to be effective^1^,^2^,^3^. In the context of motor performance, reduction of force and increase of fatigue have been described after the application of a nocebo procedure^4^,^5^,^6^, although the factors modulating this effect are still unclear, because most of the studies were carried out in the field of pain. Following new integrative models^7^, nocebo effects can be induced in different ways: with negative expectations^8^,^9^,^10^,^11^, associative learning^12^,^13^,^14^, partial conditioning^15^, social learning^16^,^17^ and even without conscious awareness and direct learning^18^. Moreover, an emerging factor in the placebo/nocebo literature is related to the way in which participants perceive the treatment in terms of choice^19^ and also in terms of its effectiveness^20^. With this regard, it was demonstrated that the discrepancy between subject’s expectation and the perception of treatment effectiveness could evoke a placebo response^20^. Beliefs about treatments and perception of treatment effectiveness have an impact in the clinical context^21^,^22^. Hence, these factors could represent an important psychological construct to be taken into account in nocebo, as well as placebo, studies in which the treatment does not have any active effect *per se*, but only simulates an active therapy in a particular psychosocial context^23^.

The current study is as an explorative investigation of the role of perception of treatment effectiveness on the nocebo effect in motor performance. More precisely, we investigated changes in perception of treatment effectiveness by adopting a conditioning procedure, in which the recipient was surreptitiously exposed to the fake effects of a treatment and implicitly learned to associate the application of the treatment to force decrements^5^. Typically, at the end of the conditioning procedure, the treatment from neutral becomes conditioned^23^,^24^ and afterwards it can elicit a nocebo response even if it is applied without any effect. The first aim of this study is to investigate whether and how strongly individuals perceive the treatment as effective when passing from the conditioning phase, in which the effect of the treatment is present, to the test session, in which the effect is removed and the nocebo response can be measured. This kind of investigation may help to better characterize individual differences in the nocebo effect in motor performance.

As we know from studies in pain perception, individuals differ in the tendency to express a nocebo response, depending on dispositional traits. Nocebo hyperalgesia is negatively predicted by optimism^25^,^26^ and positively predicted by anxiety^12^,^26^,^27^. Moreover, in the context of social observation, dispositional empathy and pain catastrophizing are associated to nocebo effects^16^,^17^. In the current explorative investigation, the second aim is to investigate whether changes in perception of treatment effectiveness could be related to some personality traits. To this purpose we assessed not only optimism (Life-Orientation Test-Revisited^28^) and anxiety (State-Trait Anxiety Inventory^29^), but also other traits that have been found to predict analgesic placebo responses^30^, like dopamine-related personality traits (i.e., harm avoidance and novelty seeking) (Temperament and Character Inventory (TCI^31^). Moreover, since the nocebo effect is induced through verbal suggestion, we assessed suggestibility (Multidimensional Iowa Suggestibility Scale^32^), in order to investigate whether the tendency to accept information from others could be associated to the subjective perception of treatment effectiveness. Finally, in order to check any change in performance due to subjective motivation in performing the motor task, we also assessed intrinsic motivation (Intrinsic Motivation Inventory^33^,^34^).

With regards to the first aim of the study, based on the evidence that perception of treatment effectiveness can influence the clinical outcome^21^, we hypothesize that individuals who perceive the treatment as strongly effective at the end of the nocebo procedure will also show a more pronounced nocebo-induced reduction of force than those who less consistently perceive its effectiveness. With regards to the second aim, based on the studies demonstrating stronger nocebo hyperalgesia in individuals with low levels of optimism and high levels of anxiety^12^,^25^,^26^,^27^, the hypothesis is that the same traits could be associated to the tendency to strongly perceive a negative effect of the treatment on motor performance.

## Methods

### Subjects

Forty-one right-handed (apart from three left-handed) healthy volunteers (18 women, mean age: 22.66 ± 3.05 years) were recruited from the student population of the University of Verona. Their performance was preliminarily compared with that of a control group made of 20 right-handed (apart from two left-handed) subjects (9 women, mean age: 21.75 ± 2.24 years), in order to confirm the validity of the paradigm in inducing a nocebo effect (see Supplementary Information and Supplementary Fig. S1). Participants self-declared to have no history of medical problems, including neurological and psychiatric disease. Before starting the study, all the subjects signed an informed consent form in which the real research aims were omitted. Only after completing the whole experimental procedure, the nocebo nature of the study was explained. The study was approved by the local ethical committee of the Department of Neurological and Movement Sciences at the University of Verona and subjects had to sign an informed consent form prior to participation. The study was conducted in accordance with the approved guidelines. Power analysis on the sample size is reported in the Supplementary Information.

### Motor task

The motor task consisted in abduction movements of the right index finger against a piston connected to a force transducer (DS BC302)^5^,^35^. The right hand was blocked by a device in order to exclude the contribution of other muscles except the first dorsal interosseous (FDI). In real-time, finger pressures against the piston were linearly converted into vertical displacements of a cursor shown on a PC monitor. In this way the subject could have a visual feedback of the amount of force. For each participant the maximum voluntary contraction (MVC) was measured before starting the experiment. This value was then used to calibrate the vertical displacements of the cursor on the PC monitor during the task. Subjects had to press the mouse with the left hand to initiate each single trial. In each trial, they were asked to press the piston with the right index finger as strongly as possible in order to bring the visible cursor into a target zone made of four colored horizontal lines which represented the 60%, 80%, 100% and 120% of the subject’s MVC. Each trial lasted 1100 ms.

### Procedure

**Figure 1:**
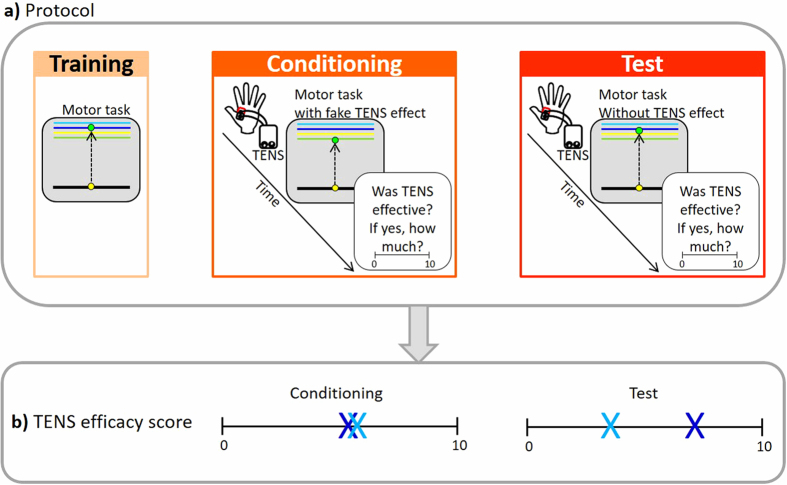
Schematic representation of the experimental protocol. (**a**) After a first training session, a conditioning session was performed in which an inert treatment (TENS) was applied together with verbal instructions that it could induce a decrease of force and with a manipulation in which the visual feedback was surreptitiously reduced. After the motor task was completed, subjects were asked to judge TENS efficacy on a VAS ranging from 0 (not effective) to 10 (very effective). Afterwards, the procedure was repeated again in the test session, but this time the (fake) effect of TENS was removed. (**b**) Example of answers of participants who gave higher scores of TENS efficacy in the test compared to the conditioning session (dark blue, positive Δ TENS effectiveness score), and participants who gave lower scores in the test compared to the conditioning (light blue, negative Δ TENS effectiveness score).

After general instructions, 5 trials were used to allow the subjects to familiarize with the instrumentation. Before starting the nocebo procedure, all the participants went through a training in which they had to perform the motor task for 50 trials (Fig. 1a). In the conditioning session, an inert treatment (10 Hz transcutaneous electrical nerve stimulation, TENS) was applied for 5 minutes over the region of the FDI muscle, together with the deceptive verbal instructions that it had the effect of reducing the recruitment of muscle fibers, with a consequent decrease in force production. The intensity of TENS was enough to produce a slight sensation on the skin, without producing muscle contraction or discomfort. Soon after TENS, participants performed the motor task (50 trials) with a surreptitious, stepwise, reduction of the cursor’s excursion range. More precisely, an attenuation coefficient was introduced and the excursion of the cursor was gradually decreased in steps of 0.0029 from trial 1 to trial 35 and then it remained stable until the end of the session (from trial 36 to trial 50). Subjects were unaware of this manipulation that was meant as a procedure to condition them about the effects of TENS in reducing force. In order to monitor subjects’ belief about TENS, we asked them to judge its efficacy on a visual analog scale (VAS) (Fig. 1a,b). More precisely, after the execution of the motor task, subjects were asked to report how much the TENS treatment was effective in reducing their force, by means of a 10 cm VAS, ranging from 0 (not effective) to 10 (very effective). In the test session, TENS was applied again with the same verbal instructions. Subjects then repeated the motor task (50 trials), but this time without any manipulation, that is without the reduction of the cursor’s excursion range. After the motor task, TENS efficacy score was measured again with the VAS (Fig. 1a,b). The difference of TENS efficacy scores between the test and conditioning sessions was taken as a proxy of the strength of perception of treatment efficacy (see in data handling for a detailed description).

#### Behavioral data

Force was evaluated through two indices. The first index was the mean value of the peak force amplitude (Force_peak_) computed in the 50 trials of each session and normalized to the MVC, as follows (1):


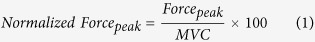


The second index regarded the percentage of strong pressures (Strong_press_) (2):


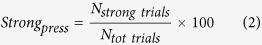


where N_tot trials_ is the total number of trials in each session (i.e., 50) and N_strong trials_ is the number of trials in which the peak force exceeded the mean value recorded in the training session. While the mean normalized force peak is a measure of the overall force level during a session, the percentage of strong pressures is a measure of consistency of behavior throughout a session.

#### Subjective data

In addition to TENS efficacy scores, other subjective variables were evaluated during the procedure: (*i*) *Perception of force*. After each session subjects had to estimate how strong they felt during the motor task by means of a 10 cm VAS scale appearing on the PC monitor, ranging from 0 (very weak) to 10 (very strong). (*ii*) *Sense of effort*. Subjective sense of effort was measured after the execution of the motor task with the Borg scale^36^, ranging from 6 (rest) to 20 (maximal effort). (*iii*) *Expectation*. Soon after the removal of TENS, before starting the motor task, subjects were asked to judge how they expected their performance will be. This judgment was given on a 7-points Likert scale, ranging from −3 (much worse) to +3 (much better) by taking as reference the training session. Expectation scores were measured twice, after each TENS application.

### Personality Questionnaires

After the whole experimental procedure, we administered some personality questionnaires in order to evaluate the possible role of personality traits. All the questionnaires were computerized (E-prime, version 2.0, Psychology Software Tools, Inc), in order to facilitate data collection and processing. In particular, we took into account: (*i*) *State-Trait Anxiety Inventory* (*STAI*)^29^. This is a 40-items inventory divided in two scales: STAI-I measures the level of anxiety in a precise moment and STAI-II refers to an individual’s usual anxiety tendency. In our study STAI-I was administered twice, before and after the procedure, to control for any change in the anxiety level across sessions, while STAI-II was applied only at the end of the procedure; (*ii*) *Life-Orientation Test-Revisited* (*Lot-R*)^28^. This is a 10-items scale developed to assess individual differences in generalized optimism versus pessimism. Four items are fillers and the total score is obtained by summing the answers of 6 real items; (*iii*) *Temperament and Character Inventory* (*TCI*)^31^. This 240-items questionnaire provides a personality profile in the context of a biopsychological model. It is made of four temperament and three character scales. The temperament dimensions are heritable traits and include novelty seeking (NS), harm avoidance (HA), reward dependence (RD) and persistence (P). The character dimensions may be related to different cognitive systems and include self-directedness (SD), cooperativeness (C) and self-transcendence (ST). One subject did not complete the TCI questionnaire due to recording problems; (*iv*) *Multidimensional Iowa Suggestibility Scale* (*MISS*)^32^. This is a 95-items questionnaire to measure social and psychological suggestibility defined as a tendency to accept messages from others. It is made of five suggestibility sub-scales: consumer suggestibility, persuadability, physiological suggestibility, physiological reactivity and peer conformity. To the purposes of the current study, we considered the total suggestibility score; (*v*) *Intrinsic Motivation Inventory* (*IMI*)^33^,^34^. This is a 37-items multidimensional inventory to assess participants’ subjective experience related to a target activity in laboratory experiments. It is divided in different scales assessing participant’s interest/enjoyment, perceived competence and choice, effort perceived during the laboratory activity, value/usefulness of the performed activity and pressure and tension felt during the activity. To the purposes of the current study, we considered the total intrinsic motivation score.

### Data handling and statistics

The variable of interest in our study was the perception of treatment effectiveness computed as difference between the test and the conditioning session (Δ TENS effectiveness). The two sessions (conditioning and test) were characterized by different visual feedbacks: during the conditioning session there was a surreptitious reduction of the cursor’s excursion range that should simulate the effect of TENS on force decrease. The cursor’s reduction was not applied in the test session. Hence, Δ TENS effectiveness represents whether and how strongly participants perceived the treatment as effective in the test compared to the conditioning session. This variable (Δ TENS effectiveness) was handled following two different statistical approaches.

In the first statistical approach, the polarity of the difference (positive or negative) was considered as means to define two groups (Fig. 1b). Conceptually, the two groups belong to two categories with opposite patterns of subjective perception: participants with a positive difference perceived the effect of the treatment more strongly in the final than in the conditioning session, whereas those with a negative difference were not so strongly consistent in their perception of treatment effectiveness. Based on this assumption, we aimed at characterizing the two categories in terms of sample size, gender distribution, performance at the motor task, and personality traits by adopting a mixed design with between and within factors. The hypothesis in this analysis is that the group with a positive TENS difference (indicating stronger perception of the negative effects of the treatment effectiveness in the test session) should also show a more pronounced nocebo effect in the behavioral (reduction of force) and subjective outcomes (feeling weakness and effort) than the group with a negative difference (indicating less perception in the negative effects of the treatment effectiveness in the test session). Based on studies in pain perception^26^,^27^, we also hypothesize that the group with a positive TENS difference should present higher levels of anxiety and lower levels of optimism than the groups with a negative difference.

For this analysis, first we checked that the two groups (positive vs. negative Δ TENS effectiveness) had similar MVC and motor performance (in terms of normalized Force_peak_) in the training session, by means of t-test for independent samples. Then, we analyzed the behavioral parameters (normalized Force_peak_, Strong_press_) and the subjective parameters (TENS effectiveness scores, subjective perception of force, sense of effort and expectation scores) in the two crucial experimental conditions (conditioning vs. test) by means of repeated measures analyses of variance (rmANOVAs) with Group as between-subject factor (positive vs. negative Δ TENS effectiveness) and Session as within-subject factor (conditioning vs. test). In addition to all the indices of performance described above, we computed also the difference (Δ) between the test and the conditioning sessions, as computational measure to evaluate the changes of the behavioral and subjective variables in the two groups. For each variable, the ∆ of the two groups were compared by means of t-tests for independent samples. The mean scores of the two groups at the personality questionnaires were analyzed with t-tests for independent samples. In all the analyses, post-hoc comparisons were executed by means of t-tests for paired or independent samples, using the Bonferroni correction for multiple comparisons where necessary. The level of significance was set at *p* < 0.05. All the data are expressed as mean ± s.e.m.

In a second statistical approach, we adopted a within design by considering all the subjects together and by dealing the difference in perception of treatment effectiveness as a continuum (from negative to positive values). The rationale for this approach is to prove whether changes in perception of treatment effectiveness were associated to changes in performance at the motor task and to personality traits. To this purpose, the difference in TENS effectiveness scores was correlated with the difference between the test and the conditioning sessions at the motor task (i.e., Δ normalized Force_peak_, Δ Strong_press_, Δ subjective perception of force, Δ sense of effort and Δ expectation). The hypothesis in this analysis is that more positive TENS effectiveness difference (indicating stronger perception in the negative effects of the treatment effectiveness in the test session), would be associated to a stronger nocebo effect measured as changes in the behavioral and subjective variables. Because we had a specific hypothesis about the direction of these correlations, 1-tailed test was used.

Difference of TENS effectiveness scores was correlated also with the personality variables. Although for some personality traits (like anxiety and lower levels of optimism) we could have specific hypotheses about the direction of the correlations^26^,^27^, for other traits (like intrinsic motivation and the temperament and character dimensions) it was not possible to make precise hypotheses and therefore 2-tailed test was used. No multiple comparisons were applied to the correlations, due to the explorative nature of the study.

Analyses were performed by using SPSS Statistics 21 software (SPSS Inc).

## Results

### Mixed design

Levene’s test revealed homogeneous variances of all the data, apart from feeling of force (p = 0.002), which was analyzed with non-parametric tests (Mann-Whitney test for comparisons between groups and Wilcoxon signed-ranks test for comparisons across sessions).

**Figure 2:**
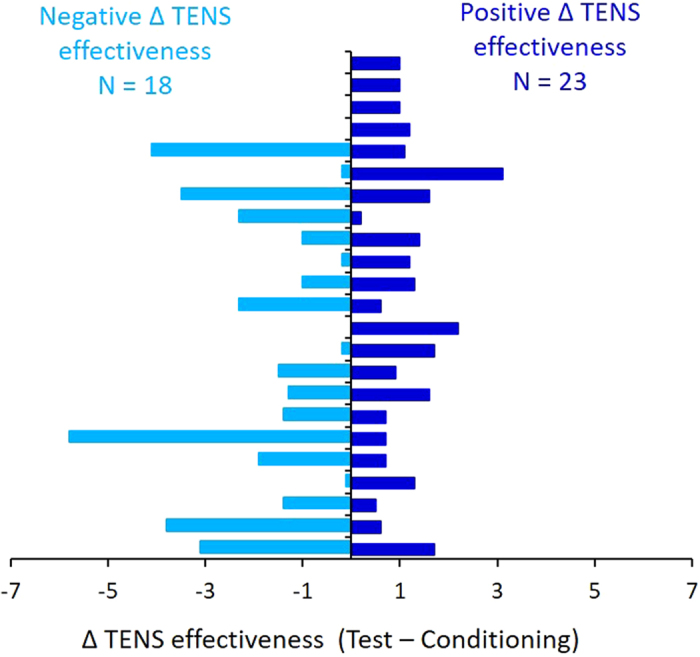
Description of the positive and negative Δ TENS effectiveness groups. By subtracting the scores of TENS efficacy in the test and conditioning sessions, it turned out that 23 participants had a positive difference (positive Δ TENS effectiveness) and 18 had a negative difference (negative Δ TENS effectiveness).

Following the first statistical approach, we found that 56.1% of the subjects (23 out of 41 subjects) had a positive difference of TENS effectiveness scores, indicating that they perceived more effect in the test than in the conditioning session (Fig. 2). Among these, 11 were females and 12 males, the mean age of the group was 22.57 ± 3.27 years and mean education was 15.3 ± 1.49 years. The remaining 43.9% of the subjects (18 out of 41) had a negative difference of TENS effectiveness scores, indicating that they perceived less effect of TENS in the test than in the conditioning session (Fig. 2). Among these, seven were females and 11 males, the mean age of the group was 22.78 ± 2.84 years and mean education was 15.29 ± 1.72 years. The two groups did not statistically differ for age (independent samples t-test, t_(39)_ = −0.218; p* *=* *0.828) and for gender distribution (Chi-square test, χ^2^ = 0.327, df = 1, p* *=* *0.567). Moreover, the two groups did not statistically differ for MVC, measured in the initial calibration phase (positive Δ TENS effectiveness: 20.53 ± 0.60 N, negative Δ TENS effectiveness: 19.41 ± 0.83 N, independent sample t-test, t_(39)_ = 1.122, p* *=* *0.269). Finally, the two groups had also similar normalized Force_peak_ in the training session (positive Δ TENS effectiveness: 89.33% ± 1.46, negative Δ TENS effectiveness: 90.1% ± 1.54, independent sample t-test, t_(39)_ = −0.351, p* *=* *0.727). These findings suggest that at the beginning of the experimental procedure, the two groups had comparable behavioral performance.

**Figure 3:**
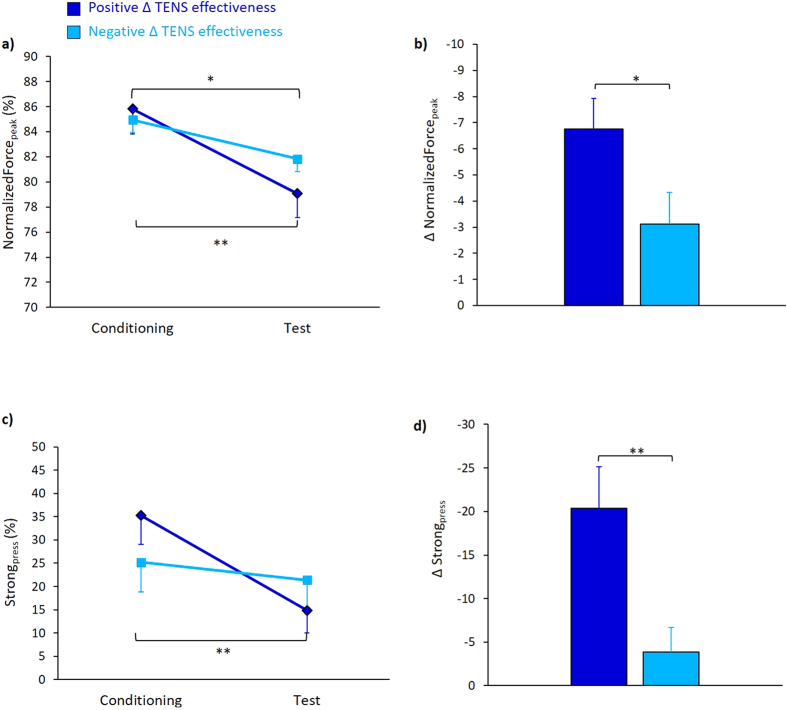
Behavioral data. (**a**) Normalized Force_peak_ of the two groups in the conditioning and test sessions. A significant decrease of force could be observed only in the group with positive Δ TENS effectiveness score. (**b**) Δ of normalized Force_peak_: the group of positive Δ TENS effectiveness score had a more marked decrease of force than the negative Δ TENS effectiveness score. The numbers on the y-axis are order from 0 to negative values. (**c**) Percentage of Strong_press_ in the two groups and in the two sessions. A significant reduction of strong pressures was found in the group of negative Δ TENS effectiveness score. (**d**) Δ of percentage of Strong_press_: the group of positive Δ TENS effectiveness score had a more marked decrease of strong pressures than negative Δ TENS effectiveness score. The numbers on the y-axis are ordered from 0 to negative values. All the data are expressed as mean values and standard errors (s.e.m.). **p < 0.01, *p < 0.05.

ANOVA on normalized Force_peak_ measured during the nocebo procedure, revealed a significant effect of Session [F_(1,39)_ = 32.65, p < 0.001, η^2^ = 0.456], due to lower values in the test (80.45% ± 1.56) compared to the conditioning (85.38% ± 1.40) session and a non-significant effect of Group [F_(1,39)_ = 0.112, p = 0.740, η^2^ = 0.003]. The interaction Session × Group was significant [F_(1,39)_ = 4.46, p = 0.041, η^2^ = 0.103]. Post-hoc comparisons showed that the group with positive Δ TENS effectiveness was weaker in test (79.06% ± 1.88) compared to the conditioning (85.81% ± 2) session (p < 0.001) and also the negative Δ TENS effectiveness group was weaker in the test (81.83% ± 2.58) compared to the conditioning (84.94% ± 1.87) session (p = 0.023) (Fig. 3a). Analysis of Δ Force_peak_ disclosed a significant difference between groups (t_(39)_ = −2.111, p = 0.041) (Fig. 3b), suggesting that positive and negative Δ TENS effectiveness groups had a different nocebo response in terms of behavioral outcome.

ANOVA on Strong_press_ disclosed a similar pattern of results with a significant effect of Session [F_(1,39)_ = 16.33, p < 0.001, η^2^ = 0.295], due to lower values in the test (18.12% ± 4.23) than in the conditioning (30.22% ± 4.55), but no effect of Group [F_(1,39)_ = 0.45, p = 0.834, η^2^ = 0.001]. The interaction Session × Group was significant [F_(1,39)_ = 7.59, p = 0.009, η^2^ = 0.163]. Post-hoc comparisons showed that the positive Δ TENS effectiveness group had a significant reduction in the percentage of strong pressure in the test (14.87% ± 4.81) compared to the conditioning (35.22% ± 6.16) session (p < 0.001), while no difference was found in the negative Δ TENS efficacy score group (p = 0.199) (Fig. 3c). Analysis of Δ Strong_press_ disclosed a significant difference between groups (t_(39)_ = −2.955, p = 0.006) (Fig. 3d). These findings confirm a different nocebo effect in performance between positive and negative Δ TENS effectiveness groups.

**Figure 4:**
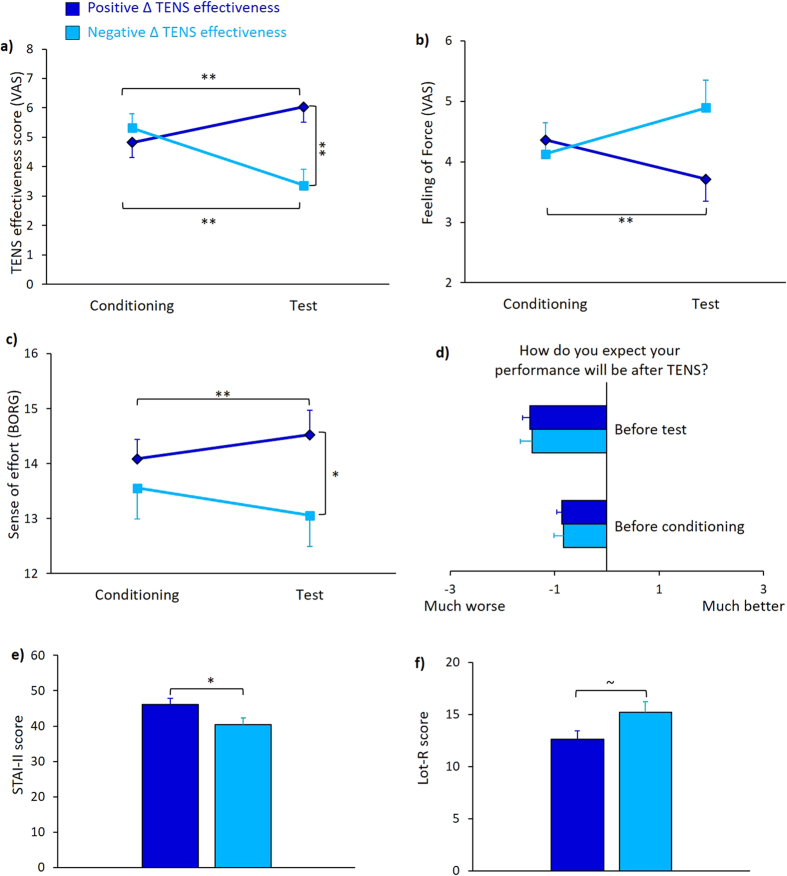
Subjective and personality data. (**a**) TENS efficacy scores of the two groups in the two sessions. In the conditioning session the two groups had similar scores of TENS efficacy, whereas in the test session the group of positive Δ TENS effectiveness score had higher scores than the group of negative Δ TENS effectiveness score. Moreover, while the group of positive Δ TENS effectiveness score had higher scores in the test compared to the conditioning session, the groups of negative Δ TENS effectiveness score had the opposite pattern, with lower scores in the test compared to the conditioning session. This suggests that the two groups were not only qualitatively, but also quantitatively different in their belief. (**b**) Subjective perception of force of the two groups in the two sessions. The group of positive Δ TENS effectiveness score felt weaker in the test compared to the conditioning session, whereas negative Δ TENS effectiveness score had a stable feeling of force. (**c**) Sense of effort at the BORG scale (Borg, 1970). The group of positive Δ TENS effectiveness score showed a significant increase of sense of effort in the test compared to the conditioning session. (**d**) Expectation of performance of the two groups in the two sessions. Expectation was measured soon after TENS application and before task execution. The scores of the two groups were not different both before the conditioning session and before the test session. (**e**) STAY II total score showed that positive Δ TENS effectiveness score had higher scores than negative Δ TENS effectiveness score. (**f**) Analysis showed a tendency of difference in total Lot-R score between positive and negative Δ TENS effectiveness score. All the data are expressed as mean values and standard errors (s.e.m.). **p < 0.01, *p < 0.05, ~*p *=* *0.051.

TENS efficacy scores were analyzed to confirm that the two groups were not only qualitative, but also quantitatively different concerning their belief in the treatment efficacy. ANOVA on TENS efficacy scores showed that on average the two groups had significantly different judgments about TENS, especially in the test session (that is in the second application of TENS). The factor Session was significant [F_(1,39)_ = 4.23, p* *=* *0.047, η^2^ = 0.098], due to lower values in the test (4.69 ± 0.38) than in the conditioning (5.07 ± 0.36) session, but no effect of Group [F_(1,39)_ = 2.27, p = 0.140, η^2^ = 0.055] was found. The Session × Group interaction was significant [F_(1,39)_ = 76.74, p < 0.001, η^2^ = 0.663]. Post-hoc comparisons showed that positive Δ TENS effectiveness group reported higher values in the test (6.03 ± 0.51) compared to the conditioning session (4.82 ± 0.51, p < 0.001), while the opposite pattern was found in the negative Δ TENS effectiveness group, with lower values in the test (3.37 ± 0.55) than in the conditioning session (5.32 ± 0.49, p < 0.001) (Fig. 4a). Moreover, the two groups had significantly different values in the test session (p = 0.001). Analysis of the Δ TENS efficacy scores confirmed a different pattern between the two groups (t_(39)_ = 7.943; p < 0.001). These findings confirm a different belief in the treatment between positive and negative Δ TENS effectiveness groups.

Wilcoxon signed-ranks test on force perception disclosed that the participants with positive Δ TENS effectiveness had lower scores in the test (3.71 ± 0.36) compared to the conditioning (4.36 ± 0.28) session (Z = −2.5, p* *=* *0.012), whereas participants with negative Δ TENS effectiveness showed no difference between sessions (Z = −1.66, p* *=* *0.098) (Fig. 4b). Mann-Whitney test on the Δ force perception showed a significant difference between the two groups (U = 96.5, Z = −2.905, p* *=* *0.004). These findings suggest that positive and negative Δ TENS effectiveness groups had a different nocebo response even in terms of subjective feeling of force.

ANOVA on the sense of effort revealed a non-significant effect of Session [F_(1,39)_ = 0.025, p* *=* *0.876, η^2^ = 0.001] and Group [F_(1,39)_ = 2.36, p* *=* *0.133, η^2^ = 0.057]. However, the Session × Group interaction was significant [F_(1,39)_ = 5.05, p* *=* *0.030, η^2^ = 0.115]. Post-hoc comparisons showed that the positive Δ TENS effectiveness group perceived a higher level of effort in the test (14.52 ± 0.45) than in the conditioning (12.52 ± 0.3) session (p < 0.001), while the negative Δ TENS effectiveness group had no differences between sessions (p = 0.217). Moreover, the two groups were different in the test session (p* *=* *0.049) (Fig. 4c). Analysis of Δ sense of effort disclosed significant differences between groups (t_(39)_ = 2.247, p* *=* *0.030). Hence, from these findings we can conclude that the sense of effort after a nocebo procedure is related to the amount of belief in the negative effects of the treatment.

Analysis of expectation scores showed that the factor Session was significant [F_(1,39)_ = 16.62, p < 0.001, η^2^ = 0.299], due to more negative values in the test (−1.45 ± 0.12) compared to the conditioning session (−0.84 ± 0.10) (Fig. 4d). Conversely, the factor Group [F_(1,39)_ = 0.04, p* *=* *0.838, η^2^ = 0.001] and the Session × Group interaction [F_(1,39)_ < 0.01, p* *=* *0.994, η^2^ = 0.000] were not significant. Moreover, the analysis of Δ expectation scores showed no significant differences between groups (p* *=* *0.994). These findings suggest that expectation was not different between positive and negative Δ TENS effectiveness groups and therefore it could be unrelated to the amount of persistence of belief in the treatment. Contingency tables on the distribution of expectation scores in the two sub-groups are reported in the Supplementary Information (see Supplementary Tables 1 and 2).

With regards to the personality traits, t-test for independent samples revealed that the positive Δ TENS effectiveness group had higher STAI-II (assessing trait anxiety) scores (46.04 ± 1.73) than the negative Δ TENS effectiveness group (40.33 ± 1.93) (t_(39)_ = 2.200, p* *=* *0.034), indicating that the former was generally more anxious than the latter (Fig. 4e).

Analysis of Lot-R (assessing optimism and pessimism) revealed a nearly significant difference between the two sub-groups (t_(39)_ = −2.012, p* *=* *0.051) with lower scores in participants with positive Δ TENS effectiveness (12.61 ± 0.83) compared to those with negative Δ TENS effectiveness (15.22 ± 1.02), suggesting that the former were more pessimist than the latter (Fig. 4f).

### Within design

**Figure 5:**
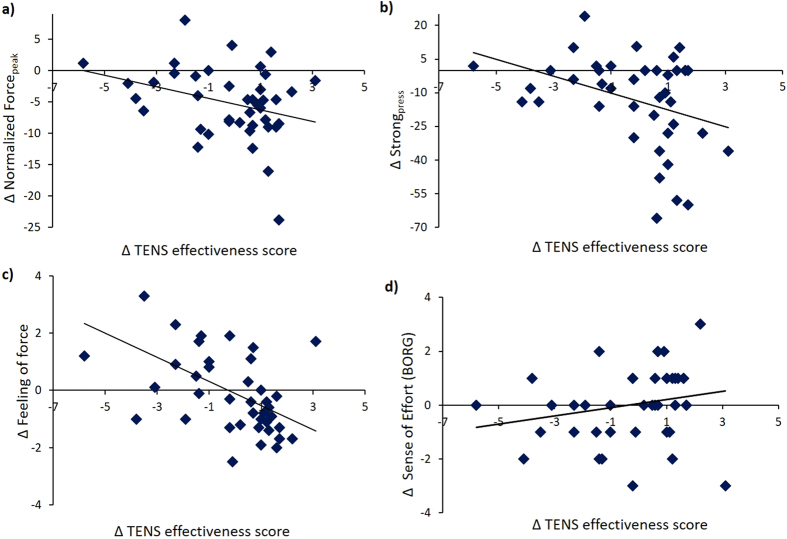
Correlations between Δ TENS efficacy scores and behavioral data. (**a**) Δ TENS effectiveness scores negatively correlated with Δ normalized Force_peak (a),_ (**b**) Δ Strong_press_ and with (**c**) Δ subjective perception of force. (**d**) Δ TENS effectiveness scores positively correlated with Δ sense of effort.

Following the second statistical approach, we found that Δ TENS effectiveness scores negatively correlated with Δ normalized Force_peak_ (rho = −0.284, p* *=* *0.036), with Δ Strong_press_ (rho = −0.282, p* *=* *0.037) and with Δ subjective perception of force (rho = −0.542, p* < *0.001) and positively correlated with Δ sense of effort (rho = 0.278, p* *=* *0.039). These findings suggest that a more positive difference in perception of TENS effectiveness was associated to a more pronounced reduction of force, reduction of feeling of force and stronger sense of effort during the nocebo procedure (Fig. 5).

**Figure 6:**
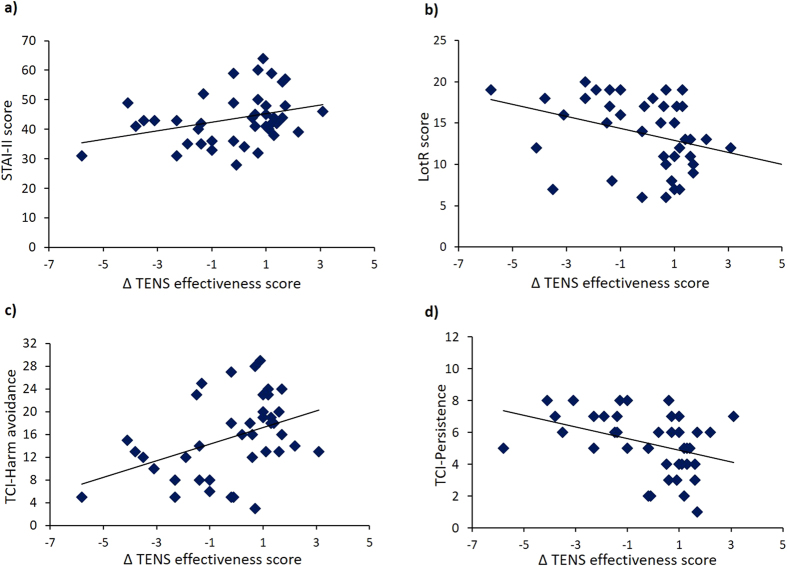
Correlations between Δ TENS efficacy scores and personality traits. (**a**) STAY-II scores positively correlated with the Δ TENS efficacy scores, suggesting that higher levels of trait anxiety were associated to higher tendency to believe in the negative effects of the treatment. (**b**) Lot-R scores negatively correlated with the Δ TENS efficacy scores, suggesting that lower levels of optimism were associated to higher tendency to believe in the negative effects of the treatment. (**c**) TCI-harm avoidance total score positively correlated with Δ TENS efficacy scores, suggesting that more inhibited, pessimist and worried individuals had a higher tendency to believe in the negative effects of the treatment. (**d**) TCI-persistence negatively correlated with Δ TENS efficacy scores, suggesting that individuals who resisted less to fatigue had a higher tendency to believe in the negative effects of the treatment.

With regards to personality traits, scores at the STAI-II positively correlated with the Δ TENS effectiveness scores (rho = 0.335, p* *=* *0.033), suggesting that the higher the anxiety trait, the stronger the perception of the negative effects of TENS in the test session (Fig. 6a).

A negative correlation with the Δ TENS effectiveness scores and Lot-R (rho = −0.373, p* *=* *0.016), suggesting that the lower dispositional optimism the stronger the perception of the negative effects of TENS in the test session (Fig. 6b). Analysis of the TCI revealed positive correlations between Δ TENS effectiveness scores and harm avoidance scale (assessing behavioral inhibition) (rho = 0.321, p* *=* *0.044) (Fig. 6c), indicating that the most inhibited individuals more strongly perceived the negative effects of TENS in the test session. A negative correlation was found with persistence (assessing perseverance in spite of fatigue or frustration) (rho = −0.367, p* *=* *0.020), suggesting that those individuals who were weaker in resisting to fatigue perceived more strongly the negative effects of TENS (Fig. 6d). No other correlations were found to be significant.

## Discussion

The findings of this study show that changes in the perception of treatment effectiveness are associated to the magnitude of the nocebo effect in motor performance and to personality traits. The same nocebo procedure, consisting of conditioning and verbal suggestion, was applied to all the participants. However, when the subjects were asked about the efficacy of the treatment in reducing force, two different patterns of response could be observed: Some subjects gave higher scores of TENS effectiveness in the test than in the conditioning session, whereas other subjects gave lower scores in the test than in the conditioning session. Of note, the two sessions were different only with respect to the surreptitious reduction of the cursor’s excursion range, which was applied in the conditioning session and removed in the test session. This manipulation served as a means to expose the participants to the effect of TENS in reducing force, thus strengthening their belief about it. In the test session, however, TENS was applied without manipulation and the pure nocebo response could be measured. Interestingly, depending on the change in perception of TENS effectiveness between the test and conditioning sessions, different nocebo responses could be observed in terms of behavioral and subjective outcomes.

With regards to the behavioral outcome, it should be first noticed that the paradigm was suitable to induce a behavioral nocebo effect, as also certified by the preliminary comparison with a control group. By dividing the subjects in relation to changes in the perception of TENS effectiveness we found that force production was differently affected in the two sub-groups. Although reduction of normalized force was observed in both sub-groups, a more pronounced decrease of percentage of strong pressures was found in individuals with a positive difference of perception of TENS effectiveness. The two indexes of performance represent slightly different aspects: Normalized force is a measure of the mean force level in relation to the MVC, whereas the percentage of strong pressures gives a measure of the consistency of behavior. Namely, the latter parameter of motor performance represents how consistently participants pressed the piston above a certain value (as determined in the training session). This finding suggests that individuals who strongly believed in the negative effects of the treatment were also less able to exert strong pressures on the piston than those who less consistently perceive the effects of the treatment. The negative correlations between changes in perception of treatment effectiveness and force reduction indicate that the more the negative effects of the treatment are perceived at the end of the nocebo procedure the stronger is the reduction of force. Overall, the behavioral results unmask a link between changes in perception of treatment effectiveness and the magnitude of the nocebo effect. The mechanisms at the basis of these findings, however, remain to be clarified in future studies.

With regards to the subjective variables, participants with a positive difference of perception of TENS effectiveness felt more weakness and effort in the test than in the conditioning session, while those with a negative difference of perception of TENS effectiveness did not modify the feeling of force and sense of effort across sessions. Interestingly, after TENS application both groups expected a similar worsening of performance. Expectation was measured twice: before the conditioning procedure and before the test session. In the first case, subjects’ expectation may have been influenced by the experimenter’s information about the treatment, prior to experience, whereas in the second case their expectation may have been influenced not only by the experimenter’s information, but also by the exposure to the effects of TENS during the conditioning phase. The fact that both groups gave similar scores at the expectation scale suggests that the procedure was successful in deceiving the subjects and that the different perception of TENS effectiveness was not triggered by different expectation levels. A challenge for future research on placebo and nocebo effects will be to investigate whether expectation and perception of treatment efficacy could be considered as two independent or interacting cognitive processes.

An interesting question in the placebo/nocebo literature is the definition of a placebo/nocebo-prone personality^37^,^38^. Differently from previous studies, we adopted a new approach, by taking into account the changes in perception of treatment effectiveness between the test and the conditioning session. The inspection of dispositional factors revealed that personality traits like anxiety and TCI-harm avoidance (which is indicative of inhibited behavior^31^,^39^) were positively correlated with the tendency to perceive the negative effects of the treatment, whereas optimism and TCI-persistence (which implies continuing and persevering despite fatigue and lack of reward^40^) were negatively correlated.

These findings well fit with the notion that optimism and anxiety modulate nocebo, as well as placebo, responses^12^,^25^,^26^,^41^. Geers and colleagues^25^ found that when participants were told that a pill would have made them feel unpleasant, pessimists were more likely than optimists to report the expected negative symptoms. Moreover, in a previous study in the context of pain, a correlation was found between nocebo responses and both anxiety scales (state and trait)^12^, supporting the link between anxiety and the nocebo effect.

In the current explorative study, we cannot make strong predictions on specific variables in being informative about other variables. Nonetheless, we could speculate that personality traits might have biased the perception of words and performance during the nocebo procedure, thus resulting in different perception of treatment effectiveness at the end of the procedure. We know from previous literature that pessimists have a stronger attentional bias toward negative stimuli^42^ and, in a similar way, high anxiety leads to be attentive to negative events^43^,^44^,^45^,^46^. Based on this evidence, we could hypothesize that more anxious and less optimistic individuals deployed more attention to the verbal information conveyed by the experimenter about the negative effects of the treatment in worsening performance. Alternatively, these individuals could have paid more attention toward the progressively shorter cursor’s excursion range, thus amplifying the perception of the negative feedback during the conditioning phase. Although the current study does not allow to disambiguate between the two alternatives, we hypothesize that these processes could have led to a stronger perception of treatment effectiveness at the end of the nocebo procedure.

Some methodological limitations of the current study should be mentioned. One limit is that we cannot draw conclusions on the causal relation between changes in perception of treatment efficacy, personality and force decrements. The division of the subjects was made *post-hoc*, based on the difference of the treatment efficacy scores between the test and the conditioning sessions. In other words, we first defined a variable to divide the two groups, i.e., the change of subjects’ perception of treatment efficacy, and afterwards we extrapolated the dispositional traits. Moreover, the mechanisms at the basis of the force decrements, sense of weakness and sense of effort remain to be uncover. Potential interest for future studies could be to manipulate expectation and perception of treatment effectiveness in a factorial design in order to investigate which factor plays a major role in the motor nocebo effect, similarly to the study by Peciña *et al*.^20^ in placebo analgesia.

Despite this unconventional way of proceeding, our study shows for the first time an association between changes in the perception of treatment effectiveness, personality traits and force production and may inspire further investigations aiming at explaining individual differences in the nocebo effect in motor performance.

## Additional Information

**How to cite this article**: Corsi, N. *et al*. Changes in perception of treatment efficacy are associated to the magnitude of the nocebo effect and to personality traits. *Sci. Rep*. **6**, 30671; doi: 10.1038/srep30671 (2016).

## Supplementary Material

Supplementary Information
