# Supplementary material for: Changes in perception of treatment efficacy are associated to the magnitude of the nocebo effect and to personality traits
Source: Sci Rep. 2016 Jul 29;6:30671. doi: 10.1038/srep30671 (PMC4965778; doi:10.1038/srep30671)
Supplement: Supplementary Information [file srep30671-s1.pdf]

Supplementary Information

Changes in perception of treatment efficacy are associated to the magnitude of the nocebo effect and to personality traits

Nicole Corsi[1,2], Mehran Emadi Andani[1,3], Michele Tinazzi[1], Mirta Fiorio[1,*]

[1] Department of Neurosciences, Biomedicine and Movement Sciences, University of Verona, Verona, 37131, Italy.

[2] Department of Pain and Translational Symptom Science, University of Maryland Baltimore, Baltimore, 21201, USA

[3] Department of Biomedical Engineering, University of Isfahan, Isfahan, 81746, Iran

***mirta.fiorio@univr.it**

Supplementary Information

Power analyses on the sample size have been conducted with G*Power3 software[1]. More precisely, assuming a priori a medium partial eta square of 0.05[2], the effect size f is equal to 0.229. Given this effect size, an α error probability of 0.05, a Power (1-β error probability) of 0.80, 2 groups (positive vs. negative Δ TENS effectiveness), 2 measurements (conditioning and final sessions), a Correlation among repeated measures of 0.5 and a nonsphericity correction ε =1, the resulting sample size is 40. To ensure an adequate sample size and prevent potential drop-outs, we recruited a total sample of 41 participants.

In order to confirm that the paradigm was suitable to induce a nocebo effect on motor performance, that is a decrease of force as shown in a previous study[3], the performance of the entire sample of subjects who underwent the nocebo procedure (nocebo group, 41 subjects, 18 women, mean age: 22.66 ± 3.05 years) was preliminarily compared with that of a control group made of 20 right-handed, but two, subjects (9 women, mean age: 21.75 ± 2.24 years). Participants of the control group underwent the same experiment, except for the nocebo procedure. More precisely, they were explicitly told that TENS was inefficient on motor performance and they executed the motor task without conditioning, that is without manipulation of the cursor.

In the statistical analysis, we first checked that the nocebo and control groups had similar force levels at the beginning of the experiment, by comparing (with t-tests for independent samples) the MVC recorded in the calibration phase and normalized $Force_{peak}$ recorded during the training. Afterwards, we compared motor performance (normalized $Force_{peak}$ and $Strong_{press}$) of the two groups in the two crucial sessions (conditioning and test) by means of repeated measures analysis of variance (rmANOVA) with Session as within-subjects factor and Group (nocebo vs. control) as between-subjects factor. Post-hoc comparisons were executed by means of t-tests for paired or

independent samples, using the Bonferroni correction for multiple comparisons where necessary and the level of significance was set at $p < 0.05$. All the data are expressed as mean ± s.e.m.

Results showed that the nocebo and control groups had similar MVC (nocebo group: 20.04 ± 0.49N, control group: 20.78 ± 0.78N, independent sample t-test, $t_{(59)} = -0.827$, $p = 0.411$) and motor performance in the training session (nocebo group: 89.66% ± 1.05, control group: 91.44% ± 2.89, independent sample t-test, $t_{(59)} = -0.579$, $p = 0.568$). ANOVA on normalized $Force_{peak}$ in the main experiment revealed a significant effect of Session ($F_{(1,59)} = 12.173$, $p = 0.001$), due to lower values in the test (84.39 ± 1.45) compared to the conditioning (87.15 ± 1.31) session. The factor Group was also significant ($F_{(1,59)} = 4.86$, $p = 0.031$), due to lower values in the nocebo group (82.86 ± 1.51) than in the control group (88.68 ± 2.17). The interaction Session × Group was significant ($F_{(1,59)} = 9.222$, $p = 0.004$). Post-hoc comparisons showed that the nocebo group was weaker in test (80.28 ± 1.54) than in the conditioning (85.3 ± 8.42) session ($p < 0.001$), while no difference was found in the control group across the two sessions ($p = 0.791$). Moreover, two groups had different values in the test session ($p = 0.006$) (see Supplementary Fig. S1). Analysis of Δ normalized $Force_{peak}$ confirmed a significant difference between groups ($t_{(59)} = -3.036$, $p = 0.004$).

ANOVA on $Strong_{press}$ disclosed no significant effect of Session ($F_{(1,59)} = 0.697$, $p = 0.407$) and no effect of Group ($F_{(1,59)} = 3.675$, $P = 0.060$), but a significant interaction Session × Group ($F_{(1,59)} = 10.126$, $p = 0.002$). Post-hoc comparisons showed that the nocebo group had a significant reduction in the percentage of strong pressure in the test (17.73 ± 4.17) compared to the conditioning (30.82 ± 4.52) session ($p < 0.001$), while no differences were found in the control group across the two sessions ($p = 0.265$). Moreover, two groups had different values in the test session ($p = 0.003$) (see Supplementary Fig. S1). Analysis of Δ $Strong_{press}$ disclosed a significant difference between groups ($t_{(59)} = -2.806$, $P = 0.009$). Altogether, these findings confirm that procedure was suitable to induce a reduction of force in the nocebo group.

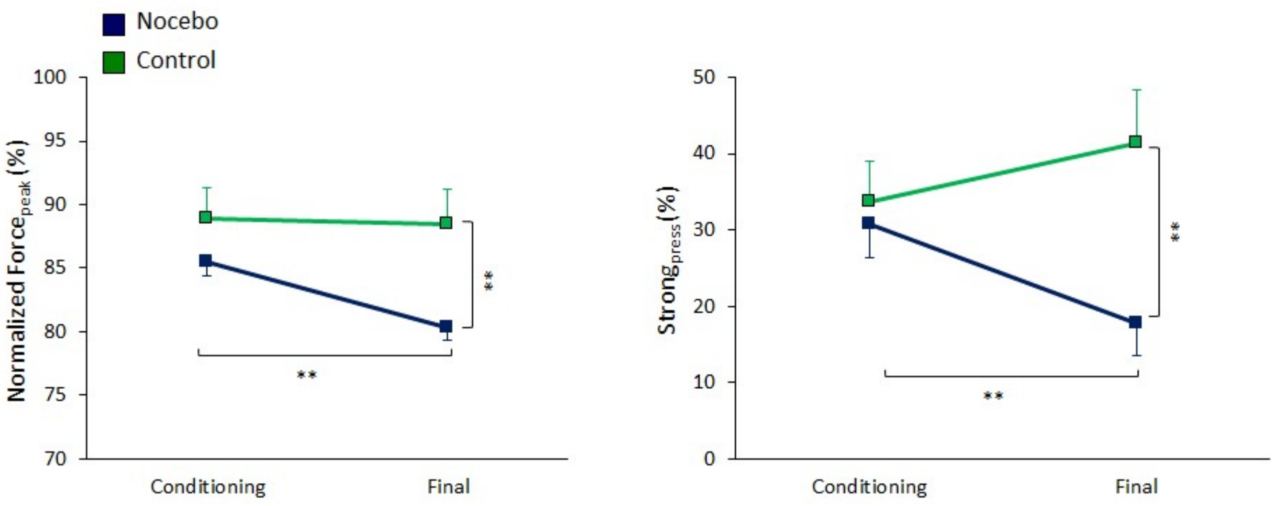

Supplementary Figure S1. **Behavioural data of the preliminary analysis. a)** Normalized Force$_{peak}$ of the two groups in the conditioning and test sessions (note that for the control group there was not a conditioning procedure, because the motor task was performed in the same way in all the sessions without manipulation of the cursor's excursion range). A significant decrease of force was observed only in the nocebo group. **b)** Percentage of Strong$_{press}$ in the two groups and in the two sessions. A significant reduction of strong pressures was found only in the nocebo group. **P < 0.01.

In order to check whether expectation scores about the effect of the treatment were equally distributed in the two nocebo sub-groups (positive vs. negative Δ TENS effectiveness), we created 2×2 contingency tables. The tables represent the expectation scores given by the two sub-groups at the first TENS application (Supplementary Table 1) and at the second TENS application (Supplementary Table 2). Expectations were categorized as negative (scores < 0 on the Likert scale) and positive/null (scores ≥ 0 on the Likert scale). Since all the subjects of the nocebo group underwent the same nocebo procedure, most of them had expectation scores < 0, indicating that

they expected a worsening in performance. We did not find differences in the distribution of the two expectation categories in the two sub-groups (Fisher's exact test: p = 0.713 for the first TENS application and p = 1.0 for the second TENS application).

**Expectation / Sub-group**	**Negative expectation**	**Positive/null expectation**	**Total**
**Positive Δ TENS effectiveness**	19	4	23
**Negative Δ TENS effectiveness**	14	4	18
**Total**	33	8	41

Supplementary Table 1. Distribution of expectation scores at the first TENS application in the two sub-groups.

**Expectation / Sub-group**	**Negative expectation**	**Positive/null expectation**	**Total**
**Positive Δ TENS effectiveness**	21	2	23
**Negative Δ TENS effectiveness**	16	2	18
**Total**	37	4	41

Supplementary Table 2. Distribution of expectation scores at the second TENS application in the two sub-groups.

References

1 Faul, F., Erdfelder, E., Lang, A. G. & Buchner, A. G*Power 3: a flexible statistical power analysis program for the social, behavioral, and biomedical sciences. *Behav Res Methods* **39**, 175-191 (2007).

2 Bakeman, R. Recommended effect size statistics for repeated measures designs. *Behav Res Methods* **37**, 379-384 (2005).

3 Emadi Andani, M., Tinazzi, M., Corsi, N. & Fiorio, M. Modulation of inhibitory corticospinal circuits induced by a nocebo procedure in motor performance. *PLoS One* **10**, e0125223, doi:10.1371/journal.pone.0125223 (2015).
